# Insulin and GH Signaling in Human Skeletal Muscle *In Vivo* following Exogenous GH Exposure: Impact of an Oral Glucose Load

**DOI:** 10.1371/journal.pone.0019392

**Published:** 2011-05-03

**Authors:** Thomas Krusenstjerna-Hafstrøm, Michael Madsen, Mikkel H. Vendelbo, Steen B. Pedersen, Jens S. Christiansen, Niels Møller, Niels Jessen, Jens O. L. Jørgensen

**Affiliations:** 1 Department of Internal Medicine and Endocrinology (MEA) and Medical Research Laboratories, Aarhus University Hospital, Aarhus, Denmark; 2 Department of Clinical Pharmacology, Aarhus University Hospital, Aarhus, Denmark; University of Padova, Medical School, Italy

## Abstract

**Introduction:**

GH induces acute insulin resistance in skeletal muscle in vivo, which in rodent models has been attributed to crosstalk between GH and insulin signaling pathways. Our objective was to characterize time course changes in signaling pathways for GH and insulin in human skeletal muscle in vivo following GH exposure in the presence and absence of an oral glucose load.

**Methods:**

Eight young men were studied in a single-blinded randomized crossover design on 3 occasions: 1) after an intravenous GH bolus 2) after an intravenous GH bolus plus an oral glucose load (OGTT), and 3) after intravenous saline plus OGTT. Muscle biopsies were taken at t = 0, 30, 60, and 120. Blood was sampled at frequent intervals for assessment of GH, insulin, glucose, and free fatty acids (FFA).

**Results:**

GH increased AUC_glucose_ after an OGTT (p<0.05) without significant changes in serum insulin levels. GH induced phosphorylation of STAT5 independently of the OGTT. Conversely, the OGTT induced acute phosphorylation of the insulin signaling proteins Akt (ser^473^ and thr^308^), and AS160.The combination of OGTT and GH suppressed Akt activation, whereas the downstream expression of AS160 was amplified by GH.

**We Concluded the Following:**

1) A physiological GH bolus activates STAT5 signaling pathways in skeletal muscle irrespective of ambient glucose and insulin levels 2) Insulin resistance induced by GH occurs without a distinct suppression of insulin signaling proteins 3) The accentuation of the glucose-stimulated activation of AS 160 by GH does however indicate a potential crosstalk between insulin and GH.

**Trial Registration:**

ClinicalTrials.gov NCT00477997

## Introduction

Growth hormone (GH) promotes longitudinal growth and somatic maturation in children and adolescents and is also an important regulator of substrate metabolism and insulin sensitivity [1]. In the post-absorptive phase, where endogenous GH secretion is stimulated, GH promotes lipolysis and oxidation of fatty acids at the expense of glucose [2],[3]. This insulin-antagonistic effect is accentuated during more prolonged fasting and may constitute a favorable protein-saving mechanism due to impeded demand for gluconeogenesis from amino acids [4]–[6]. On the other hand, sustained GH elevations in non-fasting conditions, as seen in acromegaly, may result in glucose intolerance, and manifest diabetes mellitus [7],[8].

The molecular mechanisms by which GH causes insulin resistance are unclear. Insulin-stimulated glucose transport into skeletal muscle depends on the activation of a signaling cascade involving insulin receptor substrate 1 (IRS-1), the phosphatidylinositol 3-kinase, Akt, and Akt substrate of 160 kDa (AS160) [9]. The entirety of the signaling cascade is not yet known and may include additional proteins. However, it is well known that insulin signaling ultimately promotes translocation of the glucose transporter GLUT4 to the cell surface. Any step in this cascade is a potential target for GH, and could involve direct crosstalk between signaling proteins, or indirect effects via free fatty acids (FFA), a known inhibitor of insulin receptor signaling in human skeletal muscle [10].

The predominant GH signaling cascade comprises activation of the GHR dimer, phosphorylation of JAK2 and subsequently of STAT5 [11], but there is also animal and *in vitro* evidence to suggest that insulin and GH share post-receptor signaling pathways [12]. However, a cross-talk between GH and insulin signaling pathways has not been confirmed in human models *in vivo* [13],[14]. This may, however, relate to the design of these studies. First, signaling was assessed in either the basal state, where insulin activity is minimal [14], or during a euglycemic hyperinsulinemic glucose clamp [13], which is an unphysiological condition. Second, only single biopsies were obtained in both studies, which may be insufficient because of the rapid and fluctuating nature of the post receptor signaling cascades. Third, measurement of signaling proteins downstream of Akt has so far not been performed. It should also be noted that human *in vivo* data on the time course of stimulated insulin signaling pathways after an oral glucose tolerance load have not previously been reported.

We therefore conducted a study where temporal changes in the activation of signaling proteins downstream of the receptors for GH and insulin were assessed in serial muscle biopsies in healthy human subjects following a physiological GH bolus with and without a concomitant oral glucose load (OGTT).

## Methods

### Study Protocol And Informed Consent

The study protocol was approved by The Regional Scientific Ethics Committee of Denmark (M-20070052) and all participants gave oral and written informed consent to participate. The study was conducted in accordance to the Helsinki Declaration.

### Subjects

We studied 8 healthy men aged 24.6±1.8 year (mean ± SE) with a mean body mass index of 24.2±1.2 kgxm^−2^ in a randomized, crossover design. Routine blood chemistry including fasting blood glucose and HbA1c levels were normal in all participants, none of whom received any medication.

### Study Design

**Figure 1:**
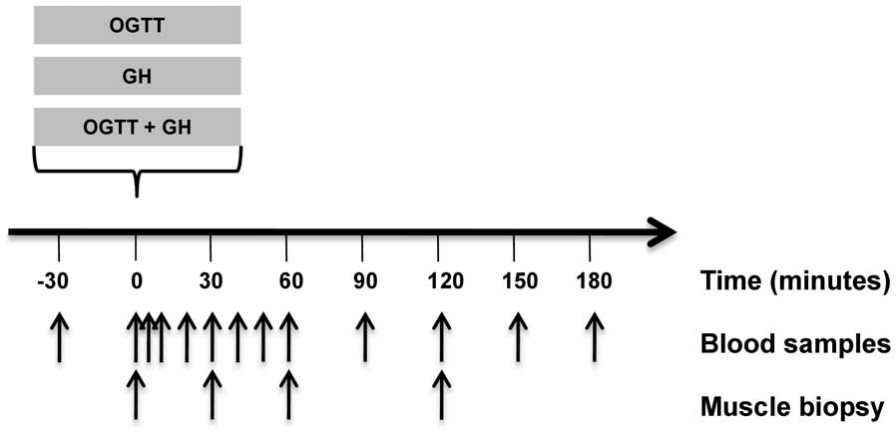
Study design. Please refer to the paragraph *study design* for further details.

Each participant was studied on 3 separate occasions in a randomized fashion (Figure 1): 1) after an intravenous GH bolus (0.5 mg Genotropin, Miniquick, Pfizer, Inc.)(GH); 2) after a blinded intravenous GH bolus (0.5 mg) plus an oral glucose load (75 g) (GH + OGTT); and 3) after a blinded intravenous saline bolus plus an oral glucose load (OGTT). At least two weeks elapsed between each study, which was performed after an overnight fast for 12 hours and with the participants resting in the supine position.

A catheter was inserted in an antecubital vein in each arm, one for administration of GH/saline, and one for blood sampling. At 09.00 h (t = 0 min) the participants received GH/saline ±OGTT. Muscle biopsies were obtained at t = 0 min (just before the intervention), t = 30 min, t = 60 min, t = 120 min. The biopsies were taken from the vastus lateralis muscle with a Bergström biopsy needle under local anesthesia (1% lidocain); a small incision was made through the skin and muscle sheath 15–20 cm above the knee. The biopsies were taken in random order two by two, meaning that the first (t = 0 min) and the second (t = 30 min) were taken from the same thigh, and the third (t = 60 min) and the fourth (t = 120 min) from the contra lateral thigh. A total amount of ≈150 mg of muscle was obtained per biopsy. The tissue was cleansed from blood (within 10 sec) and snap-frozen in liquid nitrogen. Muscle biopsies were stored at −80°C until analyzed. Blood was collected just before the first biopsy (t = 0), five min after (t = 5), and every 10 min within the first hour (t = 10, 20, 30, 40, 50, 60). After the first hour blood was collected every 30 min until one hour after the last biopsy (t = 90, 120, 150, 180). Plasma glucose and serum GH were measured at every time point. FFA was measured every 20 min within the first hour (t = 0, 20, 40, 60) and every 30 min afterwards (t = 90,120,150,180). Serum insulin was measured at 0, 20, 30, 40, 60, 120, 180 min. Body composition and aerobic exercise capacity (VO2-max) were assessed after completion of the study by Dual-emission X-ray absorptiometry and a bicycle ergometer, respectively.

### Hormones And Metabolites

Plasma glucose was measured immediately in duplicates on two Beckman Glucoanalyzers (Beckman Instruments, Palo Alto, CA). Serum insulin and GH were measured using time-resolved fluoroimmunoassays (TF-IFMA; AutoDELFIA, PerkinElmer, Turku, Finland), FFA was analyzed by a colorimetric method using a commercial kit (Wako Chemicals, Neuss, Germany).

### Intracellular Signal Transduction

Muscle biopsies were homogenized as previously described [15].

*Western blot*: Aliquots of protein were resolved by SDS-PAGE, and proteins were transferred onto nitrocellulose membranes. Immunoblotting was performed using primary antibodies as follows: phosho-STAT5, STAT5, phosho Akt, Akt2, phospho-P38, P38, phosho-Akt substrate (PAS), and Akt substrate 160 (AS160), all obtained from Cell Signaling (Beverly, MA). Membranes were incubated with horseradish peroxidase–coupled secondary antibodies, visualized by BioWest enhanced chemiluminescence (UVP LabWorks, Upland, CA) and quantified by the UVP BioImaging System.

Membranes probed with the phospho-specific antibodies were stripped in a buffer containing 100 mmolxl^−1^ 2-mercaptoethanol, 0.02 gxml^−1^ SDS and 62.5 mmolxl^−1^ Tris–HCl (pH 6.7), and re-probed with corresponding total antibody. The signal from the phospho-specific antibodies was related to total protein expression in the sample. STAT5 protein bands were identified using human muscle stimulated with GH as positive controls [14].The remaining bands were identified using insulin stimulated rat muscle [16]. Phosphorylation of AS160 was identified as insulin responsive band at approximately 160 kDa using the phospho-Akt substrate (PAS) antibody (Cell Signaling). This antibody has been shown to primarily identify AS160 in human skeletal muscle [17],[18] but a potential cross-reaction with the AS160 paralogue TBC1D1 (∼155 kDa) cannot be completely excluded.

### Isolation Of Rna

Skeletal muscle (20 mg) was homogenized in TriZol reagent (Gibco BRL, Life Technologies, Roskilde, Denmark). RNA was quantitated by measuring absorbency at 260 nm and 280 nm and the Integrity of the RNA was checked by visual inspection of the two ribosomal RNAs on an ethidium bromide stained agarose gel.

### Real-Time Rt-Pcr For Mrna Analysis

Reverse transcription was performed using random hexamer primers as described by the manufacturer (GeneAmp RNA PCR Kit from Perkin Elmer Cetus, Norwalk, CT). Then, PCR-mastermix containing the specific primers and Taq DNA polymerase (HotStar Taq, Quiagen Inc. USA) were added. The following primers were designed using the primer analysis software Oligo version 6.64:

IGF1: 5′GACAGGGGCTTTTATTTCAAC 3′and 5′ CTCCAGCCTCCTTAGATCAC 3′, 117 bp, SOCS1: 5′ACACGCACTTCCGCACATTC 3′and 5′ CGAGGCCATCTTCACGCTAAG 3′, 209 bp; SOCS2: 5′GGTCGAGGCGATCAGTG 3′and 5′ TCCTTGAAGTCAGTGCGAATC 3′, 209 bp; SOCS3: 5′CGGCCACTTGGACTCTGA 3′and 5′ GCCCTTTGCGCCCTTT 3′, 106 bp; β-actin 5′ ACGGGGTCACCCACACTGTGC 3′ and 5′ CTAGAAGCATTTGCGGTGGACGATG 3′, 658 bp. Real time quantization of target gene to ß-actin mRNA was performed with a SYBR-Green real-time PCR assay using an ICycler from BioRad. The threshold cycle (Ct) was calculated, and the relative gene-expression was calculated essentially as described in the User Bulletin #2, 1997 from Perkin Elmer (Perkin Elmer Cetus, Norwalk, CT).

### Statistics

Data are presented as means ± SE when normally distributed, and median (ranges) (25%; 75%) when not. Statistical evaluation of differences between normally distributed data was performed with a paired *t*-test and with Wilcoxon rank sum test when data were not normally distributed. Time series of serum measurements and results from Western blots were analyzed by ANOVA for repeated measurements or by using area under curve (AUC). Correlation analyses were performed using Person's correlation coefficient. A p value<0.05 was considered statistical significant. Statistical analysis was performed using SPSS version 17.0 for windows (SPSS, Chicago, IL).

## Results

### Glucose

**Figure 2:**
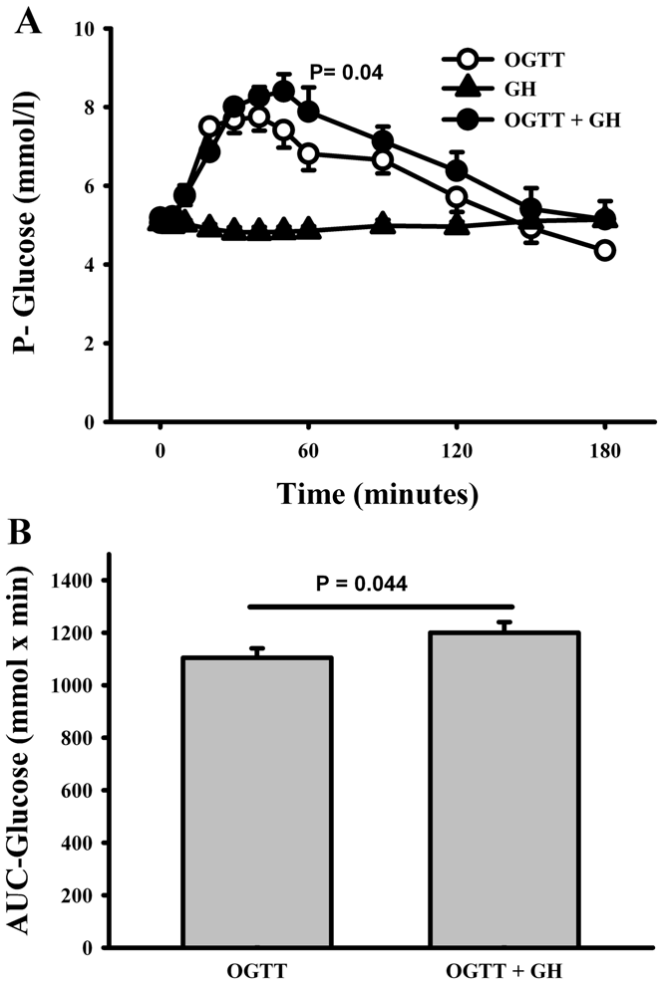
Glucose measurements. (A) Plasma levels of glucose. OGTT, oral glucose tolerance test (75 g glucose). GH, growth hormone bolus (0.5 mg). Black circles = OGTT + GH, white circles = OGTT, black triangles = GH. Data are presented as mean ± SE. Using ANOVA repeated measurements showed a significant difference between OGTT and OGTT+GH (p = 4) (B) AUC-glucose, area under glucose curve. P-value is based on paired t-test between area under curve for OGTT and area under curve for OGTT+GH. Data are presented as mean ± SE. There was a significant difference in AUC-glucose between OGTT and OGTT+GH (p = 0.04).

A significant difference between the plasma glucose curves obtained from OGTT+GH vs. OGTT alone was recorded (ANOVA, p = 0.04) (Figure 2a). There also was a statistical significant difference in AUC_glucose_ ((mmolxl^−1^xmin^−1^) [1200±41 (OGTT+GH) vs. 1105±35 (OGTT) (p = 0.04) (Figure 2b)]. Moreover, GH together with an OGTT tended to increase peak levels of plasma glucose (C_max_) (p = 0.06) compared to OGTT alone (Figure 2a).

### Insulin, Gh, And Ffa

**Figure 3:**
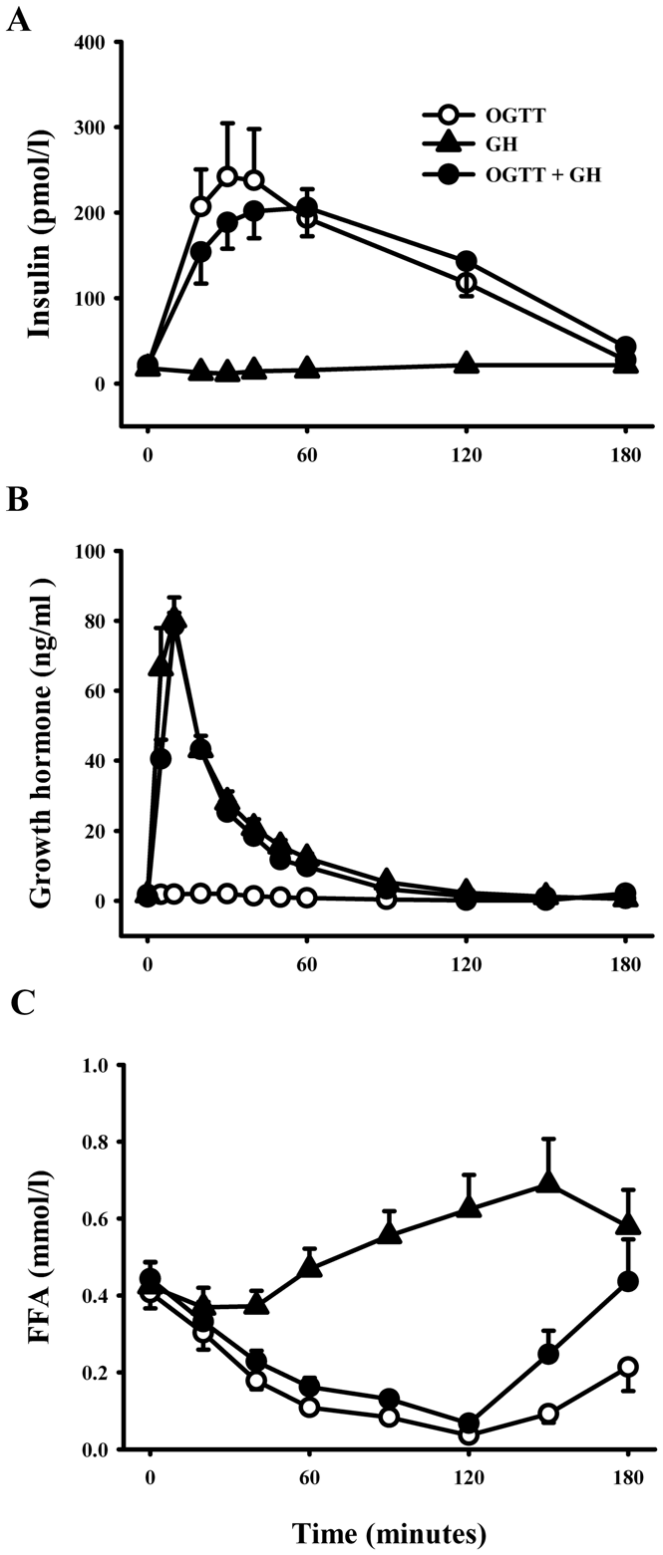
Hormones and metabolites. (A) Serum levels of insulin, no significant difference between OGTT and GH+OGTT could be recorded using ANOVA repeated measurements (p = 0.51) or AUC_insulin_ (p = 0.84). (B) Growth Hormone, (C) FFA, the degree of FFA suppression was identical throughout the first 120 min between OGTT and GH + OGTT but the assessed the presence of GH after 120 min caused a reversal of the insulin suppression of lipolysis which made AUC_FFA_ differ significantly. Black circles = OGTT + GH, white circles = OGTT, black triangles = GH. Data are presented as mean ± SE.

Baseline and glucose-stimulated insulin levels (pmolxl^−1^) were not significantly influenced by concomitant GH exposure [C_max_: 281±57 (OGTT) vs. 243±33 (OGTT+GH) (p = 0.39); T_max_ (min): 49±11 (OGTT) vs. 61±14 (OGTT+GH) (p = 0.57) (Figure 3a)]. No significant difference in insulin patterns as a function of time and treatment between OGTT and GH+OGTT could be recorded (ANOVA, p = 0.51). Likewise, we did not observe a difference in AUC_insulin_ ((pmolxl^−1^xmin^−1^) between OGTT and OGTT+GH [24908±4769 (OGTT) vs. 25523±4633 (OGTT+GH) (p = 0.843)]. This suggests that the muscles were stimulated by equal amounts of insulin in the two situations.

The GH bolus yielded serum GH peak values after 10 min without any impact of a concomitant OGTT (p = 0.81) (Figure 3b). Likewise, a comparable log-linear decline in serum GH levels was recorded when comparing GH and GH+OGTT.

GH induced a ≈60% increase in serum FFA levels after 150 min, which was followed by a gradual decline towards baseline levels after 3 hours (Figure 3c). This lipolytic effect of GH was suppressed by the concomitant OGTT as characterized by a ≈85% suppression after 120 min and a subsequent increase towards baseline levels after 3 hours. As expected, OGTT alone induced a pronounced ≈90% decrease in serum FFA levels after 120 min followed by a minor increase after 3 hours to a level still ≈50% lower than baseline. The degree of FFA suppression was identical throughout the first 120 min between OGTT and GH+OGTT assessed by AUC_FFA_ (p = 0.083), however the presence of GH after 120 min caused a reversal of the insulin suppression of lipolysis which made AUC_FFA_ differ significantly (p = 0.026).

### Stat5

**Figure 4:**
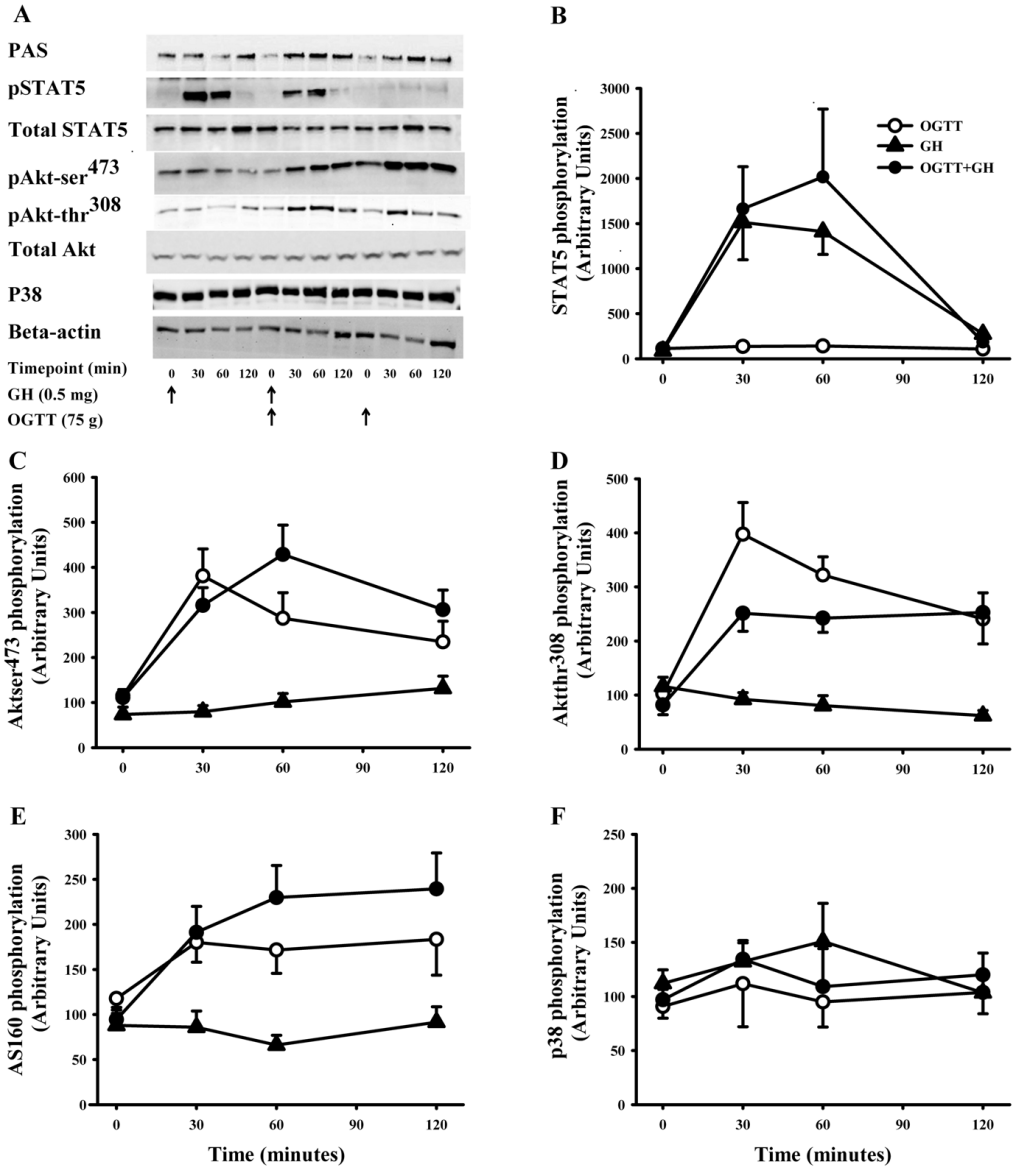
Western blot data. (A) Western blots illustrating comparable levels of phosphorylated insulin signaling proteins (PAS, Aktser^473^, Aktthr^308^, P38, pSTAT5, total STAT5 and total Akt). Arrows indicate exposure. Effects of a GH bolus (0.5 mg) and/or an OGTT (75 g) on phosphorylation of (B) STAT5. GH induced phosphorylation of STAT5 independently of the OGTT (C) Aktser^473^, (D) Aktthr^308^, (E) AS160, and (F) P38. Black circles = OGTT + GH, white circles = OGTT, black triangles = GH. Data are presented as mean ± SE.

GH induced a significant 17.5-fold increase in pSTAT5 (AU) after 30 min compared to baseline. At 60 min the increase was 16- fold and at 120 min 3- fold, still significantly increased compared to baseline [87±16 (baseline) *vs.* 1513±415 (30 min) (p = 0.014); *vs.* 1412±254 (60 min) (p = 0.002); *vs.* 275±65 (120 min.) (p = 0.027)]. The same pattern was recorded when GH was combined with OGTT (Figure 4a and 4b). ANOVA for repeated measurements showed no significant difference in pSTAT5 between GH and GH+OGTT (p = 0.64). The OGTT alone did not impact pSTAT5 (Figure 4a). We did not detect any differences in total STAT5 expression as a function of either time or treatment. To summarize, GH induced phosphorylation of STAT5 independently of the OGTT.

### Akt

The OGTT induced a significant increase in phosphorylation of Akt at ser^473^ and thr^308^ (AU), which was detectable at 30 min., 60 min and 120 min compared to baseline (Figure 4a, 4c, and 4d). A similar pattern was recorded when OGTT was combined with GH exposure, although the most pronounced increase in phosphorylation of Akt at ser^473^ occurred after 60 min rather than 30 min and phosphorylation of Akt at thr^308^ was significantly lower at t = 30 min (p = 0.049) and at t = 60 min (p = 0.03). By contrast GH alone did not induce significant changes in phosphorylation of Akt at either site. Using ANOVA for repeated measurements we found no statistical significant difference between the two curves (OGTT and OGTT+GH) for either Aktser^473^ (p = 0.56) or Aktthr^308^ (p = 0.15). Phosphorylation of Akt at both ser^473^ and thr^308^ was positively correlated to insulin levels (p<0.001).We did not detect any changes in total Akt protein expression as a function of either time or treatment.

### As160 (tbc1d4) And P38

Baseline PAS phosphorylation of AS160 using the phospho-Akt substrate antibody was comparable on all study days. OGTT alone induced a significant increase in AS160 PAS phosphorylation (AU) after 30 min [117±13 (baseline) *vs.* 180±22 (30 min), p = 0.013], which was followed by non significant elevated levels at 60 and 120 min compared to baseline (Figure 4a, 4e). OGTT+GH induced a more pronounced increase in PAS phosphorylation of AS160 (AU) which was significant at 30 min, 60 min, and 120 min compared to baseline (OGTT+GH: 94±14 (baseline) *vs.* 191±29 (AU) (t = 30 min) (p = 0.014) *vs.* 230±36 (t = 60 min) (p = 0.011) *vs.* 240±40 (t = 120 min) (p = 0.007). ANOVA showed no significant difference between OGTT and GH+OGTT (p = 0.39). GH exposure alone induced a decrease in PAS phosphorylation of AS160 after 60 min. [88±13 (baseline) vs. 66±11 (60 min), p = 0.049], followed by a return to baseline levels (Figure 4d). There was a positive correlation between AS160 PAS phosphorylation and insulin (r = 0.86, p<0.000). As regards P38 no significant changes were recorded with time in either experiment (Figure 4f).

### Igf-I And Socs1–3 Mrna Expression

Muscle biopsies taken at 0 min and 120 min were used for the analysis of IGF-I and SOCS1–3 mRNA expression. No significant increase in the expression of IGF-I mRNA was observed in any of the experiments (Table 1). By contrast, GH alone significantly increased the expression of SOCS-2 mRNA (AU) (p = 0.008), and SOCS-3 mRNA (AU) (p = 0.005] compared to baseline. GH + OGTT significantly increased the expression of SOCS-1 mRNA (AU) (p = 0.03), SOCS-2 mRNA (AU) (p = 0.015), and SOCS-3 mRNA (AU) (p = 0.038) compared to baseline. There was no significant expression of SOCS1–3 mRNA when oral glucose was given alone.

**Table 1:** IGF-1 and SOCS 1–3 mRNA.

mRNA	Units	Event	Time/min	Median	(25%;75%)	Test
**IGF-I**	AU	OGTT	0	2.41	(0.99;11.5)	
	AU	OGTT	120	1.34	(0.16;9.88)	P = 0.11
	AU	GH	0	7.50	(2.05;15.5)	
	AU	GH	120	6.26	(2.38;13.3)	P = 0.38
	AU	OGTT+GH	0	10.5	(2.30;25.1)	
	AU	OGTT+GH	120	12.4	(3.02;28.3)	P = 0.11
**SOCS-1**	AU	OGTT	0	0.55	(0.37;1.66)	
	AU	OGTT	120	1.40	(0.30;1.52)	P = 1.00
	AU	GH	0	0.32	(0.12;1.21)	
	AU	GH	120	1.79	(0.55;14.9)	P = 0.13
	AU	OGTT+GH	0	0.43	(0.24;0.86)	
	AU	OGTT+GH	120	2.71	(1.16;3.34)	P = 0.03*
**SOCS-2**	AU	OGTT	0	0.55	(0.50;1.50)	
	AU	OGTT	120	0.83	(0.31;1.98)	P = 0.56
	AU	GH	0	1.21	(0.43;1.73)	
	AU	GH	120	3.07	(1.75;3.97)	P = 0.01*
	AU	OGTT+GH	0	1.31	(0.39;2.20)	
	AU	OGTT+GH	120	5.26	(2.29;15.2)	P = 0.02*
**SOCS-3**	AU	OGTT	0	0.67	(0.65;0.99)	
	AU	OGTT	120	2.76	(0.51;3.10)	P = 0.22
	AU	GH	0	0.89	(0.52;1.25)	
	AU	GH	120	4.41	(2.02;5.89)	P = 0.01*
	AU	OGTT+GH	0	2.06	(0.59;3.87)	
	AU	OGTT+GH	120	4.67	(2.91;15.3)	P = 0.04*

P-value after Wilcoxon rank sum test.

GH, growth hormone; OGTT,oral glucose tolerance test; AU, arbitrary unit.

doi:10.1371/journal.pone.0019392.t001

### Correlations

To assess the impact of body composition and physical fitness on GH signaling the percentage of total body fat (%) and lean body mass (%) were correlated to peak levels of pSTAT5 and SOCS mRNA expression during the GH-only study. Significant positive correlations were found between TBF and pSTAT5 (r = 0.79, p = 0.037), SOCS-2 (r = 0.79, p = 0.020) and SOCS-3(r = 0.80, p = 0.016). Significant negative correlations were found between LBM and pSTAT5 (r = −0.79, p = 0.033), SOCS-2 (r = −0.79, p = 0.019) and SOCS-3(r = −0.80, p = 0.016), and between VO_2_- max/kg and pSTAT5 (r = −0.76, p = 0.05) and SOCS-3 m RNA (r = −0.73, p = 0.04), respectively.

## Discussion

It is well documented that GH acutely induces insulin resistance in human skeletal muscle in vivo [2],[19]–[22], but the underlying molecular mechanisms remain unknown. In particular - and in contrast to animal data [12] - studies in human models have not been able to document an inhibitory effect of GH on insulin signaling pathways in either muscle or fat [1],[13],[14],[23],[24].The human studies, however, have been conducted either in the basal state or during a hyperinsulinemic glucose clamp, neither of which reflects the physiological condition of a meal-induced stimulation of endogenous insulin secretion and action. In the present study we therefore exposed healthy subjects to an oral glucose load in the absence and presence of acute concomitant GH exposure. This was accompanied by serial muscle biopsies to measure time course changes in pertinent GH and insulin signaling proteins.

We observed that exposure to a single GH bolus translated into transient activation of STAT5 signaling in skeletal muscle, which was uninfluenced by a concomitant oral glucose load. Conversely, the oral glucose load stimulated insulin signaling in skeletal muscle, which was modified but not abrogated by concomitant GH exposure.

The present study confirms that phosphorylation of STAT5 in skeletal muscle is a very robust and reproducible effect of systemic GH exposure in human subjects [13],[14],[23],[25], and it demonstrates for the first time that activation of STAT5 peaks 60 min after a GH bolus followed by a decline towards baseline levels after 120 min. In support of a physiological role of this response, it is noteworthy that endogenous GH stimulated by either ghrelin [24] or exercise [26] also induces pSTAT5 in human skeletal muscle in vivo. It is likely that the signaling response to an exogenous GH bolus is influenced by the participant's pre-study exposure to GH. Recognized determinants of GH secretion and action in human subjects include age, gender, body composition and physical fitness [27],[28]. We observed a positive correlation between the participants TBF and GH signaling, whereas both LBM and VO_2_- max/body weight correlated negatively with GH signaling. Fat mass is known to be inversely related to GH secretion (also in normal weight subjects), whereas the opposite is true for LBM and VO_2_- max [27],[28]. To reconcile these observations we speculate that pre-study GH levels may suppress GH signaling induced by an exogenous GH bolus. This hypothesis obviously needs to be experimentally addressed in future studies which also should account for other determinants of GH secretion such as gender and age.

The GH-induced activation of STAT5 was unaffected by a concomitant oral glucose load, which is in accord with observations made during a hyperinsulinemic glucose clamp [13]. It has previously been reported that prolonged (8–24 h) but not short-term (4 h) insulin pretreatment inhibits GH signaling via the GHR/JAK2/STAT5B pathway in rat hepatoma cells [29],[30]. Conversely, rapid tyrosine phosphorylation of STAT5 by insulin has been recorded in a perfused rat liver model [31]. Whether these discrepancies reflect tissue-specific or species-specific differences remain uncertain, but at present there is no evidence to support that insulin interacts with GH signaling in human muscle or fat in vivo.

We observed that insulin signaling proteins in human skeletal muscle in vivo are activated in a distinct temporal pattern within 30 min after an OGTT. The serial measurements of insulin signaling activity during the OGTT allow examination of temporal physiological changes that may not be detected during a glucose clamp. Muscle glucose uptake is difficult to quantify directly during an OGTT. However it has previously been demonstrated that glucose from an OGTT for the most part is disposed into skeletal muscle [32]. We therefore consider an OGTT an acceptable model for studying the impact of GH on stimulated insulin signaling and glucose uptake in skeletal muscle.

Previous studies in human in vivo models have failed to detect effects of GH, given as either an infusion [23] or a bolus [13],[14], on insulin signaling via IRS-1 associated PI3-kinase [14],[23], serine/threonine kinase Akt [13],[14],[23], and Erk1 [13]. This, together with the present data, deviates from animal as well as *in vitro* studies showing that inhibition of the IRS1-Akt pathway is a mechanism whereby GH induces insulin resistance in skeletal muscle [12] and fat [33]. Our present data, however, show that phosphorylation of the intermediary signaling proteins Aktser^473^ and Aktthr^308^ tended to be delayed (Aktser^473^) and suppressed (Aktthr^308^) when GH was given in combination with OGTT, whereas further downstream phosphorylation of AS160 was more pronounced when GH was combined with OGTT. Most agree that phosphorylation of Aktthr^308^ occurs prior to phosphorylation of Aktser473 and that this is a two-step process; based on our data it is likely that GH may interact with this process. The physiological significance, however, is unclear when considering that the activation of AS160, which is downstream of Aktser473, was activated rather than suppressed by GH. It remains to be studied whether the latter may reflect an inhibitory effect of GH on insulin signaling downstream of AS160.

It is well known that GH via STAT5 stimulates SOCS expression [34] and that SOCS-3 is the major negative regulator of GH signaling [35]–[38]. Animal studies suggest that SOCS-1 inhibits insulin-stimulated activation of the Erk1/2 and Akt *in vivo*, and phosphorylation of IRS-1 by the IR *in vitro* [39]. We found that after 2 hours, GH induced a significant increase in the expression of both SOCS2 and SOCS3 mRNA expression, and that GH in combination with OGTT also induced a significant increase in SOCS1 mRNA. However, none of these changes was associated with the phosphorylation of Akt. It is also well described that elevated FFA levels are causally linked to insulin resistance although the underlying mechanisms are unclear [10],[40],[41]. In accordance with this, we have previously observed that experimental suppression of lipolysis in conjunction with GH administration in GH-deficient adults significantly abrogates the antagonistic effects of GH on insulin-stimulated muscle glucose uptake [22], and that insulin resistance induced by short-term high dose GH administration in healthy adults is accompanied by accumulation of fat in muscle cells [42]. But in contrast to data obtained with intralipid infusion in human subjects, we have not been able to detect suppression of either PI 3-kinase or Akt/PKB following GH-induced insulin resistance during a glucose clamp despite a marked elevation in circulating FFA levels [23]. In the present study the lipolytic effect of GH was blunted by the concomitant OGTT, although the degree of suppression was significantly less as compared to OGTT alone (Figure 3c). Measurement of intramyocellular lipid content would have strengthened the study but would have required a separate preparation and thus much larger biopsies.

### Conclusions

We conclude that a physiological GH bolus activates STAT5 signaling pathways acutely in skeletal muscle irrespective of ambient circulating glucose and insulin levels. The acute antagonistic effects of GH on glucose-stimulated insulin action were accompanied by a moderate suppression of Akt activation, whereas the expression of the more downstream signaling protein AS160 was amplified rather than suppressed by GH. Our model provides a viable tool to study GH and insulin action in human target tissues in vivo.
